# Small molecule inhibitors of WNT/β-catenin signaling block IL-1β- and TNFα-induced cartilage degradation

**DOI:** 10.1186/ar4273

**Published:** 2013-08-21

**Authors:** Ellie BM Landman, Razvan L Miclea, Clemens A van Blitterswijk, Marcel Karperien

**Affiliations:** 1 Department of Developmental BioEngineering, MIRA Institute for Biomedical Technology and Technical Medicine, University of Twente, Drienerlolaan 5, 7522 NB Enschede, the Netherlands; 2 Department of Pediatrics, Leiden University Medical Centre, Einthovenweg 20, 2333 ZC Leiden, the Netherlands; 3 Department of Tissue Regeneration, MIRA Institute for Biomedical Technology and Technical Medicine, University of Twente, Drienerlolaan 5, 7522 NB Enschede, the Netherlands

## Abstract

**Introduction:**

In this study, we tested the ability of small molecule inhibitors of WNT/β-catenin signaling to block interleukin 1β (IL-1β)- and tumor necrosis factor α (TNFα)-induced cartilage degradation. Proinflammatory cytokines such as IL-1β and TNFα are potent inducers of cartilage degradation by upregulating matrix metalloproteinase (MMP) expression and activity. Because WNT/β-catenin signaling was found to be involved in IL-1β- and TNFα-induced upregulation of MMP activity, we hypothesized that inhibition of WNT/β-catenin signaling might block IL-1β- and TNFα-induced cartilage degradation. We tested the effect of small molecules that block the interaction between β-catenin and TCF/Lef transcription factors on IL-1β- and TNFα-induced cartilage degradation in mouse fetal metatarsals.

**Methods:**

We used mouse fetal metatarsals treated with IL-1β and TNFα as an *ex vivo *model for cytokine-induced cartilage degradation. Metatarsals were treated with IL-1β and TNFα in combination with the small molecules PKF115-584, PKF118-310 and CGP049090 at different concentrations and then harvested them for histological and gene expression analysis.

**Results:**

We found that IL-1β- and TNFα-induced cartilage degradation in mouse fetal metatarsals was blocked by inhibiting WNT/β-catenin signaling using small molecule PKF115-584 and partially using CGP049090 dose-dependently. In addition, we found that PKF115-584 blocked IL-1β- and TNFα-induced MMP mRNA expression, but did not reverse the inhibitory effect of IL-1β on the expression of cartilage anabolic genes.

**Conclusion:**

In this study, we show that inhibition of WNT/β-catenin signaling by small molecules can effectively prevent IL-1β- and TNFα-induced cartilage degradation by blocking MMP expression and activity. Furthermore, we elucidate the involvement of WNT/β-catenin signaling in IL-1β- and TNFα-induced cartilage degradation.

## Introduction

In degenerative cartilage diseases such as osteoarthritis (OA) and rheumatoid arthritis (RA), the balance between anabolic and catabolic processes is shifted toward breakdown of the extracellular cartilage matrix [1-3]. Cartilage destruction is thought to be the result of increased expression and activity of catabolic proteins, such as matrix metalloproteinases (MMPs) [4]. Expression of *MMP1 *(collagenase), *MMP3 *(stromelysin), *MMP9 *(gelatinase) and *MMP13 *(collagenase 3) mRNA has been found in chondrocytes in arthritic cartilage [5,6]. Increased mRNA expression of *MMP1 *and *MMP3 *was also found in the synovial tissue of OA patients [7]. In agreement with that finding, protein expression of MMP1, MMP3 and MMP9 in the synovial fluid of patients with OA in the temporomandibular joint was found to be increased compared to healthy control joints [8]. The essential role of MMPs in cartilage degradation was illustrated by experimental evidence indicating that *Mmp13*-deficient mice were resistant to cartilage damage in medial meniscus destabilization-induced cartilage degradation [9]. In addition, cartilage degradation induced by IL-1β and oncostatin M in human and bovine articular cartilage explants could be blocked by a specific MMP13 inhibitor [10].

Proinflammatory cytokines such as interleukin (IL)-1β and tumor necrosis factor α (TNFα) potently induce MMP expression and activity in cartilage, and these cytokines are associated with cartilage degradation *in vitro *and *in vivo *[6,11,12]. The increased expression of several MMPs in human articular cartilage explants in similar locations where IL-1β and TNFα were highly expressed is suggestive of the involvement of IL-1β and TNFα in the stimulation of MMP expression [11]. *In vitro *and *in vivo *studies have shown that proinflammatory cytokines such as IL-1β and TNFα are present in both OA and RA joint tissues and synovial fluid [1,4,13]. IL-1β is associated with cartilage degeneration, whereas TNFα was shown to be involved in driving inflammation [3]. Besides their role in cartilage degradation by stimulating MMPs, IL-1β and TNFα impair the ability of the cartilage to restore the extracellular matrix by blocking the synthesis of new extracellular matrix components [3].

Recently, the canonical WNT/β-catenin signaling pathway in the pathophysiology of cartilage degenerative disease has attracted much attention [14]. The WNT/β-catenin signaling pathway is activated upon binding of WNT to its receptor Frizzled (FZD) and coactivator low-density lipoprotein receptor-related protein 5 (LRP5)/LRP6. Subsequently, the degradation complex for β-catenin is destabilized, resulting in high cytoplasmic levels of β-catenin and translocation of β-catenin to the nucleus, where it binds to transcription factor/lymphoid enhancer-binding factor (TCF/Lef), leading to activation of target genes [15]. Several lines of evidence predominantly derived from animal models support the involvement of WNT/β-catenin signaling in the molecular mechanism underlying cartilage degradation. Conditional activation of β-catenin in articular chondrocytes in adult mice was found to result in articular cartilage destruction with accelerated terminal chondrocyte differentiation [16]. It has also been shown that knockout of *FRZB*, an antagonist of canonical WNT signaling makes mice more susceptible to chemically induced articular cartilage degradation [17]. Furthermore, increased expression of secreted FZD-related proteins, which prevents binding of WNTs to their receptors, was found in OA synovium, which might be indicative of a compensatory mechanism for increased WNT signaling [18].

Recently, a link between WNT/β-catenin signaling and IL-1β-induced cartilage degradation was found. Expression of WNT5a and WNT7a in articular chondrocytes was induced by IL-1β [19], and the combination of IL-1β and WNT3a induced greater loss of proteoglycans from the extracellular matrix than either one alone [12]. In addition, induction of WNT signaling by either recombinant WNT3a or glycogen synthase kinase 3β (GSK3β) inhibitor 6-bromoindirubin 3'-oxime (BIO) was shown to induce MMP mRNA expression and proteolytic activity in mouse cartilage explants. The fact that knockdown of TCF4 eliminated this effect indicates the involvement of TCF4 in WNT-induced MMP expression [20]. In addition, involvement of Lef1 was found in increased MMP13 expression upon IL-1β stimulation [21].

Because proinflammatory cytokine-induced cartilage degradation appears to involve WNT/β-catenin signaling and increased WNT/β-catenin signaling has been implicated in the initiation and progressive deterioration of cartilage degeneration, we hypothesized that small molecule inhibitors of the interaction between β-catenin and TCF4 and Lef1 could be used to prevent cytokine-induced cartilage degradation. The aim of this study was to assess the potential effects of small molecules that inhibit the WNT/β-catenin signaling pathway on the degeneration of cartilage. We have selected the small molecules PKF115-584, PKF118-310 and CGP049090, which block the binding of β-catenin to its transcription factor TCF4. PKF115-584 and CGP049090 also block the binding between β-catenin and transcription factor Lef1 [22-24]. In addition, PKF115-584 not only blocks but also disrupts the binding between β-catenin and TCF [22-24]. To study the potential effect of these WNT inhibitors, we used explanted mouse fetal metatarsals, in which we induced cartilage degradation by adding IL-1β and TNFα.

## Materials and methods

### Luciferase assay

HEK-293t cells were seeded at 7,500 cells/cm^2 ^into 96-well plates (Nalge Nunc International, Penfield, NY, USA) and cultured for 24 h in Dulbecco's modified Eagle's medium supplemented with 10% fetal bovine serum (FBS) and 100 U of penicillin-streptomycin (Gibco/Life Technologies, Grand Island, NY, USA) prior to transfection with the TOPflash TCF/Lef luciferase reporter construct (Upstate Biotechnology/EMD Millipore, Lake Placid, NY, USA) and pRL-CMV control vector (Promega, Madison, WI, USA). Cells were stimulated with the GSK3β inhibitor BIO (Sigma-Aldrich, St Louis, MO, USA) to stimulate the WNT/β-catenin pathway in combination with the inhibitors 24 h after transfection. After 24 h of stimulation, luminescence was measured using the Dual-Glo Luciferase Assay System (Promega).

### Metabolic activity

To study the effect of small molecules on metabolic activity, the preosteoblast cell line KS483-4C3 was used [25]. Twenty-four hours after seeding, KS483-4C3 cells were stimulated with different concentrations of the compounds, and, 24 h later, the metabolic activity was determined using the 3-(4,5-dimethylthiazol-2-yl)-2,5-diphenyltetrazolium bromide (MTT) assay. Cells were incubated with MTT for 4 h, and, after stopping the reaction by adding dimethyl sulfoxide, optical density was measured at 540 nm.

### Immunofluorescence staining for nuclear accumulation of β-catenin

Nuclear accumulation of β-catenin was detected by immunofluorescence staining. KS483-4C3 cells were seeded onto glass slides (Nalgene Nunc International) and treated with LiCl, both with and without small molecule inhibitors. After 3 h, cells were washed in phosphate-buffered saline and fixed in 3.7% buffered formalin. Subsequently, cells were quenched in 50 mM NH_4_Cl for 10 min and incubated overnight at 4°C in NET GEL buffer (50 mM Tris, pH 7.4, 150 mM NaCl, 5 mM ethylenediaminetetraacetic acid, 0.05% Nonidet P-40, 0.25% gelatin and 0.02% azide). The next day cells were incubated with anti-β-catenin antibody (1:500 in NET GEL buffer; BD Transduction Laboratories, San Jose, CA, USA) for 1 h at room temperature. Next, cells were incubated with anti-mouse fluorescein isothiocyanate-labeled secondary antibody (1:250 in NET GEL buffer; Sigma-Aldrich) for 1 h at room temperature and mounted using VECTASHIELD mounting medium (VECTOR Laboratories, Burlingame, CA, USA).

### Mouse fetal metatarsals

Mouse fetal metatarsals were isolated from FVB mouse embryos (time-paired; Harlan Laboratories, Indianapolis, IN, USA) at day 17.5 of gestation [26,27]. After isolation, metatarsals were individually cultured in 24-well plates in 200 µl/well in Minimal Essential Medium (MEM) α supplemented with 10% FBS, 100 U of penicillin-streptomycin (Gibco/Life Technologies) and 1% GlutaMAX supplement (Invitrogen, Carlsbad, CA, USA) for 48 h. After this equilibration period, metatarsals were treated with several concentrations of the small molecules, either alone or in combination with 10 ng/ml TNFα or IL-1β (R&D Systems, Minneapolis, MN, USA) or a combination of both for 1, 4 or 7 days. Animal experiments were approved by the ethical committee of the University Medical Centre Utrecht.

### Morphometric and histological analysis

Optical microscopy was performed at different time points, and the lengths of the metatarsals were measured along the sagittal axis of the bone using ImageJ image analysis software (National Institutes of Health, Bethesda, MD, USA). For histological examination, metatarsals were fixed in 10% formalin and dehydrated in ethanol series before being embedded in paraffin. Five-micrometer sections were cut using a rotary microtome (HM 355S; Microm International, Walldorf, Germany). Sections were stained for glycosaminoglycans using 0.5% Alcian Blue (Sigma-Aldrich) in H_2_O (pH set to 1 using HCl) for 30 min and counterstained in 1% Nuclear Fast Red solution (Sigma-Aldrich) for 5 min. For immunohistochemical staining of collagen type II, sections were preincubated in 5 µg/ml proteinase K (Sigma-Aldrich) for 10 min, followed by 1 mg/ml hyaluronidase (Sigma-Aldrich) for 30 min, both at 37°C. Rabbit polyclonal collagen type II primary antibody (Abnova, Jhongli City, Taiwan) was diluted 1:1,000 and incubated overnight at 4°C. For visualization, the EnVision+ HRP kit (Dako, Carpinteria, CA, USA) was used.

### Gene expression analysis

Five metatarsals were pooled and lysed in TRIzol reagent (Ambion/Life Technologies, Austin, TX, USA) for RNA isolation, using the NucleoSpin RNA II kit (Macherey-Nagel, Düren, Germany) according to the manufacturer's protocol. Subsequently, cDNA was synthesized using the iScript cDNA Synthesis Kit (Bio-Rad Laboratories, Hercules, CA, USA). Quantitative polymerase chain reaction was performed using iQ SYBR Green Supermix (Bio-Rad Laboratories) in the MyiQ 2 Two-Color Real-Time PCR Detection System (Bio-Rad Laboratories). Gene expression was normalized using glyceraldehyde 3-phosphate dehydrogenase and expressed as fold change compared to controls. Primer sequences are listed in Table 1.

**Table 1:** Primer sequences for quantitative polymerase chain reaction

Gene name	Primer sequence	Product size	Annealing temperature
*ACAN*	Forward: 5'-AGGCAGCGTGATCCTTACC-3' Reverse: 5'-GGCCTCTCCAGTCTCATTCTC-3'	136 bp	60°C

*COL2A1*	Forward: 5'-CGTCCAGATGACCTTCCTACG-3' Reverse: 5'-TGAGCAGGGCCTTCTTGAG-3'	122 bp	60°C

*SOX9*	Forward: 5'-TGGGCAAGCTCTGGAGACTTC-3' Reverse: 5'-ATCCGGGTGGTCCTTCTTGTG-3'	98 bp	60°C

*MMP3*	Forward: 5'-TGGCATTCAGTCCCTCTATGG-3' Reverse: 5'-AGGACAAAGCAGGATCACAGTT-3'	116 bp	60°C

*MMP9*	Forward: 5'-GGTGATTGACGACGCCTTTGC-3' Reverse: 5' CGCGACACCAAACTGGATGAC 3'	115 bp	60°C

*MMP13*	Forward: 5'-AAGGAGCATGGCGACTTCT-3' Reverse: 5'-TGGCCCAGGAGGAAAAGC-3'	72 bp	60°C

*GAPDH*	Forward: 5'-CGCTCTCTGCTCCTCCTGTT-3' Reverse: 5'-CCATGGTGTCTGAGCGATGT-3'	82 bp	60°C

*B2M*	Forward: 5'-GACTTGTCTTTCAGCAAGGA-3' Reverse: 5'-ACAAAGTCACATGGTTCACA-3'	106 bp	60°C

### Statistical analysis

The results are expressed as mean values with 95% confidence intervals (CIs), and statistical significance was tested using analysis of variance with PASW Statistics 18 software (SPSS, Inc, Chicago, IL, USA).

## Results

### Effect of small molecules on TCF/Lef reporter activity, nuclear translocation of β-catenin and metabolic activity

**Figure 1:**
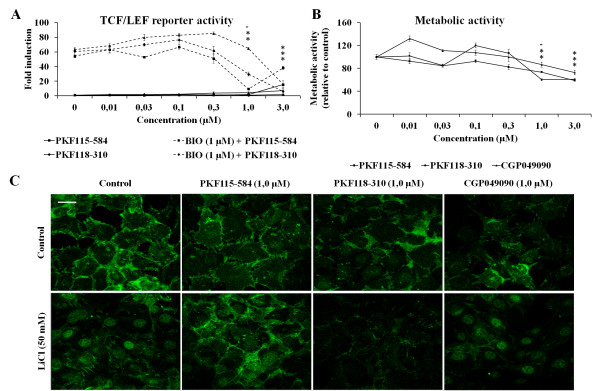
**Small molecule inhibitors of WNT/β-catenin signaling effectively block TCF/Lef-mediated activity of β-catenin**. **(A) **Small molecules dose-dependently inhibit transcription factor/lymphoid enhancer-binding factor (TCF/LEF) reporter activity in HEK-293t cells, induced by the glycogen synthase kinase 3β (GSK3β) inhibitor 6-bromoindirubin 3'-oxime (BIO) (1.0 µM). Data represent the means of three independent experiments with 95% confidence intervals (CIs). **(B) **Metabolic activity, measured using a 3-(4,5-dimethylthiazol-2-yl)-2,5-diphenyltetrazolium bromide assay in KS483-4C3 cells, was not affected by small molecules at lower concentrations; however, at 1.0 µM (except for CGP049090) and 3.0 µM, metabolic activity was significantly decreased. Data represent the means of three independent experiments with 95% CI. **(C) **Treatment with 50 mM LiCl induced nuclear translocation of β-catenin. Small molecules by themselves had no effect on cellular localization of β-catenin, whereas PKF118-310 and PKF115-584 blocked LiCl-induced translocation of β-catenin to the nucleus. CGP049090 did not affect nuclear accumulation of β-catenin after LiCl treatment. A representative example of three independent experiments is shown. Scale bar represents 10 µm *P < 0.05 placed in the order relative to the order of the data points below..

We first tested the efficacy and specificity of the small molecule compounds to inhibit canonical WNT/β-catenin signaling in HEK-293t cells transiently transfected with the TOPflash TCF/Lef luciferase reporter construct. The compounds were tested in the presence or absence of BIO, a potent activator of WNT/β-catenin signaling by blocking GSK3β [28]. We found a dose-dependent decrease in reporter activity when the cells were treated with BIO and the small molecule inhibitors. PKF115-584 treatment resulted in a sixfold decrease in reporter activity at a concentration of 1.0 µM. At a concentration of 3.0 µM, luciferase reporter activity increased again, most likely due to notable cell death upon visual inspection of the cultures. PKF118-310 and CGP049090 were slightly less effective. Maximal inhibition was found at 3.0 µM, which decreased reporter activity by fourfold and sixfold, respectively (Figure 1A).

The effect of small molecules on the metabolic activity of cells was tested in KS483-4C3 cells using an MTT assay [25]. No significant effects on metabolic activity were found when cells were treated with lower concentrations of the compound; however, 1.0 µM or 3.0 µM PKF115-584 and PKF118-310 and 3.0 µM CGP049090 did cause a significant decrease in metabolic activity (Figure 1B).

Because β-catenin can effectively activate the WNT/β-catenin pathway only after nuclear translocation, the cellular localization of β-catenin was determined by immunofluorescence staining. Figure 1C shows that stimulation of the WNT/β-catenin pathway by LiCl, which also inhibits GSK3β, resulted in translocation and accumulation of β-catenin in the nucleus. At 1.0 µM, CGP049090 reduced the intensity of β-catenin staining but did not inhibit nuclear translocation induced by LiCl, whereas PKF118-310 reduced the intensity of β-catenin staining and also inhibited LiCl-induced nuclear translocation. In contrast, PKF115-584 did not affect the intensity of the β-catenin staining under basal conditions. In the presence of LiCl, β-catenin membrane staining was increased, but nuclear translocation was markedly inhibited by PKF115-584.

### Tumor necrosis factor α and interleukin 1β induce cartilage degradation in mouse fetal metatarsals

**Figure 2:**
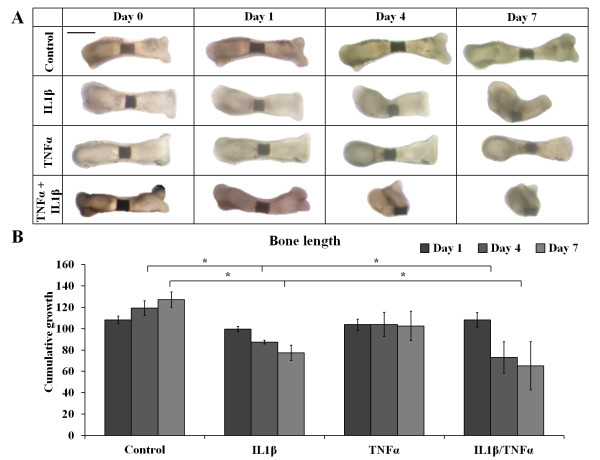
**Combined treatment with interleukin 1β and tumor necrosis factor α caused cartilage degradation in mouse fetal metatarsals**. **(A) **Mouse fetal metatarsals treated with a combination of interleukin 1β (IL-1β) and tumor necrosis factor α (TNFα) exhibit abundant cartilage resorption, whereas treatment with IL-1β alone had minor effects and TNFα tended to blunt longitudinal growth only. A representative picture of six independent experiments is shown. Scale bar represents 500 µm. **(B) **Treatment with IL-1β or a combination of IL-1β and TNFα significantly decreased bone length after 4 days and 7 days of treatment. Data represent the means of six independent experiments with 95% confidence intervals. *P < 0.05).

Metatarsals were cultured in medium containing IL-1β or TNFα or a combination of both at concentrations of 10 ng/ml. TNFα tended to blunt longitudinal bone growth, although this did not reach significance. In contrast, IL-1β alone induced bone and cartilage resorption, resulting in a significant reduction in bone length after 4 and 7 days of treatment. Cotreatment of IL-1β with TNFα caused even more abundant bone and cartilage resorption, resulting in a significant reduction in bone length (Figures 2A and 2B). Because IL-1β and TNFα together were more effective than either IL-1β or TNFα alone, we used the combination of IL-1β and TNFα to induce cartilage degradation in further experiments.

### Inhibitory effect of small molecules on cartilage degradation in mouse fetal metatarsals

**Figure 3:**
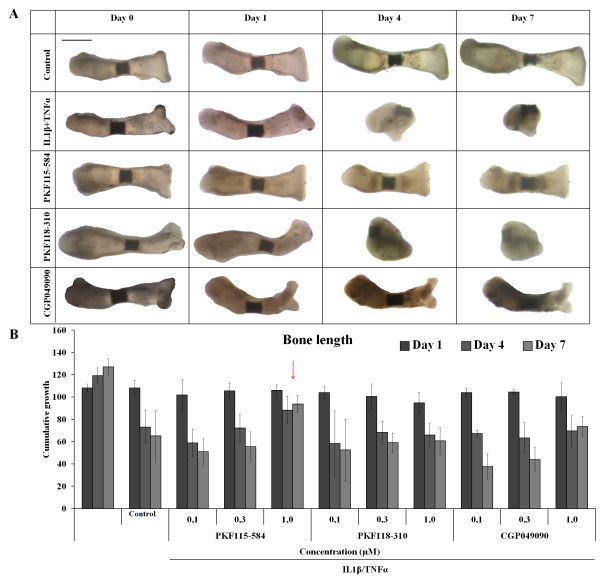
**Cartilage degradation induced by interleukin 1β and tumor necrosis factor α in mouse fetal metatarsals can be blocked by small molecule WNT inhibitors**. **(A) **Morphological changes of metatarsals caused by interleukin 1β (IL-1β) and tumor necrosis factor α (TNFα) (10 ng/ml each) can be blocked by cotreatment with PKF115-584 at a concentration of 1.0 µM. CGP049090 partially blocks resorption of the metatarsals, whereas PKF118-310 did not have an effect. A representative picture of three independent experiments is shown. Scale bar represents 500 µm. **(B) **PKF115-584 dose-dependently blocked a decrease in bone length caused by IL-1β/TNFα treatment (10 ng/ml each) over time (indicated by red arrow). Other compounds and other concentrations did not counteract detrimental effects of IL-1β/TNFα treatment. Data represent the means of three independent experiments with 95% confidence intervals.

Because visual inspection of cell cultures treated with concentrations higher than 1.0 µM revealed increased cell death, most likely due to toxic side effects, we chose to test the effect of the three WNT inhibitors at a dose range from 0.1 µM to 1.0 µM. When metatarsals were treated with the small molecules only, no effect on the *in vitro *growth of the metatarsals was observed at any of the concentrations that were tested (Additional file 1: Figure S1). The decrease in growth and resorption of the metatarsals when treated with TNFα and IL-1β was counteracted when metatarsals were treated with small molecules. At a concentration of 1.0 µM, PKF115-584 blocked bone and cartilage resorption most effectively. Also, CGP049090 counteracted the detrimental effects of TNFα and IL-1β on explant resorption, albeit less effectively than PKF115-584, whereas PKF118-310 had no significant effect (Figure 3A).

**Figure 4:**
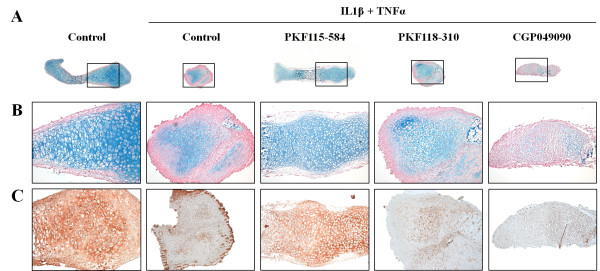
**Interleukin 1β/tumor necrosis factor α-induced loss of glycosaminoglycans and collagen II was blocked by cotreatment with PKF115-584**. **(A) **Metatarsals were treated with interleukin 1β (IL-1β) and tumor necrosis factor α (TNFα) (10 ng/ml each) in combination with small molecule WNT/β-catenin inhibitors (1.0 µM). PKF115-584 preserved morphology and glycosaminoglycan staining. A representative picture of two independent experiments is shown. Scale bar represents 500 µm. Boxed areas represent areas represented in B. **(B) **Magnification of the boxed region in (A). Scale bar represents 100 µm. **(C) **After 7 days of treatment, PKF115-584 (1.0 µM) prevented IL-1β and TNFα (10 ng/ml each)-induced loss of collagen II staining. A representative picture of three independent experiments is shown. Scale bar represents 100 µm.

Alcian Blue staining for glycosaminoglycans demonstrated that the glycosaminoglycan content of the cartilaginous matrix of IL-1β/TNFα-treated metatarsals was decreased. In line with the effect of IL-1β and TNFα on glycosaminoglycans in the extracellular matrix, Alcian Blue staining was partially preserved by cotreatment with PKF115-584 and PKF118-310, but not with CGP049090 (Figures 4A and 4B). Also, collagen II staining of the extracellular matrix was completely lacking after treatment with IL-1β and TNFα, whereas cotreatment with PKF115-584, but not with PKF118-310 or CGP049090, could prevent loss of collagen II from the extracellular matrix (Figure 4C).

### Small molecule WNT/β-catenin inhibitors decrease expression of matrix catabolic genes

**Figure 5:**
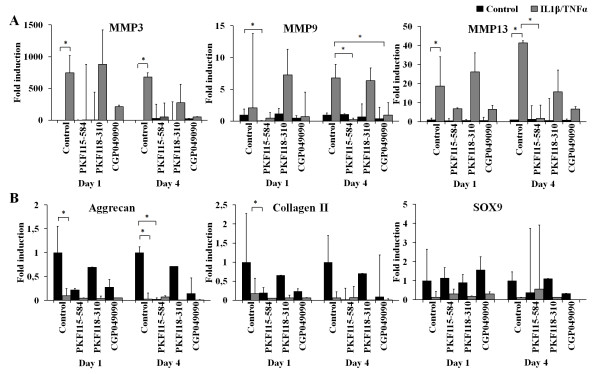
**Small molecule inhibitors block interleukin 1β (IL-1β)/tumor necrosis factor α (TNFα)-induced expression of matrix metalloproteinases without affecting the IL-1β/TNFα-induced decrease in mRNA expression of cartilage markers**. **(A) **Significant upregulation of *Mmp3 *expression was found when metatarsals were treated with IL-1β and TNFα. Both PKF115-584 and CGP049090 decreased this upregulation after 4 days of cotreatment, whereas PKF118-310 did not have an effect. *Mmp9 *expression was significantly downregulated by PKF115-584 after 1 day and IL-1β/TNFα-induced upregulation was prevented by both PKF115-584 and CGP049090, but not by PKF118-310, after 4 days of culture. Expression of *Mmp13 *was significantly upregulated by IL-1β and TNFα, whereas this effect was blocked by cotreatment with PKF115-584 or CGP049090, but not with PKF118-310. MMP, matrix metalloproteinase. **(B) ***Acan *expression is significantly decreased by IL-1β and TNFα at both day 1 and day 4. Also, small molecules PKF115-584 and CGP049090 downregulated expression of *Acan *by themselves. Expression of *Col2a1 *is downregulated by IL-1β and TNFα, and this effect could not be counteracted by small molecules. No significant effects on *Sox9 *expression were found. Data represent the means of two independent experiments with 95% confidence intervals. *P < 0.05.

Because the catabolic effect of cytokines on cartilage consists of both induction of MMP expression and downregulated expression of cartilage matrix genes, we tested the effect of small molecule inhibitors on mRNA expression of these genes. In line with the findings reported in previous studies [6,12], IL-1β and TNFα significantly induced expression of *Mmp3*, *Mmp9 *and *Mmp13*. Small molecule PKF115-584 significantly downregulated IL-1β/TNFα-induced expression of *Mmp9 *and *Mmp13 *after 4 days of treatment, whereas CGP049090 only blocked the IL-1β/TNFα-induced upregulation of *Mmp9 *after 4 days (Figures 5A and 5B). The expression of cartilage matrix genes *Acan *and *Col2a1 *was significantly downregulated, and the expression of *Sox9 *tended to decrease, upon treatment with IL-1β and TNFα after 1 and 4 days of treatment. PKF115-584 and CGP049090, and to a lesser extent PKF118-310, also decreased the mRNA expression of *Acan *and *Col2a1 *from day 1 forward. Neither compound was able to counteract IL-1β/TNFα-induced reduction in gene expression neither at day 1 nor at day 4. The three inhibitors did not affect *Sox9 *or counteract the effect of IL-1β/TNFα on *Sox9 *expression after 1 day of treatment. Prolonged treatment with small molecules, with the exception of PKF118-310, decreased *Sox9 *expression.

## Discussion

We hypothesized that inhibition of WNT/β-catenin signaling in cartilage might be an effective therapeutic strategy for the treatment of cytokine induced cartilage degradation. Therefore, in this study we have tested this hypothesis by assessing the effect of recently identified small molecule inhibitors of WNT/β-catenin signaling on cartilage degradation in the absence or presence of the pro-inflammatory cytokines IL-1β and TNFα, which are known to potently stimulate cartilage degradation by upregulating the expression of MMPs and aggrecanases [1,11].

Using the TOPflash TCF/Lef luciferase reporter construct experiments, we have shown that the small molecule inhibitors effectively block WNT/β-catenin signaling while having only a minor unfavorable effect on the metabolic activity of KS483-4C3 cells at higher concentrations as measured by MTT assay. Immunofluorescence staining for β-catenin revealed that PKF115-584 blocked nuclear translocation of β-catenin upon LiCl stimulation, without altering total β-catenin expression in basal conditions or after stimulation. PKF118-310 and CGP049090 slightly decreased the expression of β-catenin under basal conditions as well as upon LiCl stimulation. PKF118-310 blocked β-catenin translocation after LiCl stimulation, whereas CGP049090 did not affect nuclear translocation of β-catenin. Taking these findings together, we conclude that the small molecule inhibitors we selected can be used in further experiments to assess the effect on *in vitro *cartilage degradation. The discrepancy in the effect of the different small molecule inhibitors on nuclear translocation of β-catenin might indicate different mechanisms of action between these compounds.

To study the effects of the compounds on IL-1β- and TNFα-induced cartilage degradation, we used an *ex vivo *model consisting of mouse fetal metatarsals [29,30]. In degenerative cartilage disease, not only chondrocytes but also osteoblasts in the underlying bone are involved. The organ culture system that we used includes the primary center of ossification and the developing bone collar, as well as the cartilage template, providing chondrocytes as well as osteoblasts. Previously, it has also been shown that, in this model, system immune cells, including macrophages and osteoclasts, reside in the perichondrium [31]. This allows for communication between different cell types implemented in degenerative joint diseases that cannot be mimicked in other *in vitro *models, such as cartilage explants. Furthermore, in this system, chondrocytes and osteoblasts are in their natural environment, allowing the different cell types to interact with each other and with the extracellular matrix as they would *in vivo*. In line with the findings reported in previous studies, we observed that IL-1β and TNFα are potent inducers of cartilage and bone degradation in mouse fetal metatarsals [32-34]. Therefore, we considered this model to be suitable for studying the effect of small molecule inhibitors of the WNT/β-catenin signaling pathway on cartilage degradation induced by proinflammatory cytokines. In line with data reported in the literature, IL-1β alone demonstrated a mild effect on explant degradation. TNFα did not have an effect, but acted synergistically with IL-1β [29]. We therefore have chosen the combination of these cytokines to induce explant degradation and to evaluate the potential effect of the WNT/β-catenin inhibitors. Cartilage degradation is mainly due to increased expression and activity of MMPs, which can be induced by, among others, IL-1β and TNFα [1,11]. Indications of the involvement of WNT/β-catenin in IL-1β/TNFα-induced upregulation of MMPs have been found previously [12]. On the basis of morphometric and histological examination, we have shown that inhibition of WNT/β-catenin signaling by PKF115-584 can prevent the catabolic effects of IL-1β and TNFα on cartilage. CGP049090 prevented degradation of the extracellular matrix as well, albeit less effectively, whereas PKF118-310 did not have an anticatabolic effect.

Gene expression analysis revealed that the compounds, particularly PKF115-584 and CGP049090, inhibit IL-1β/TNFα-induced expression of catabolic genes *Mmp3, Mmp9 *and *Mmp13*. This indicates that inhibition of WNT/β-catenin signaling has an anticatabolic effect by blocking the induction of MMPs by IL-1β and TNFα. In line with the findings reported in previous studies [12], this observation further indicates that WNT/β-catenin signaling is involved in IL-1β/TNFα-induced MMP expression. As mentioned above, the catabolic effect of inflammatory cytokines consists, on the one hand, of the increased expression and activity of matrix-degrading proteins and, on the other hand, of decreased expression of cartilage anabolic genes. For the WNT/β-catenin inhibitors to block cartilage degradation effectively, they should interfere with both components of cartilage destruction. We found that small molecule inhibitors do block the catabolic process induced by IL-1β and TNFα. However, we did not find an effect of WNT/β-catenin inhibition on recovery of basal gene expression levels of extracellular matrix components *Acan *and *Col2a1 *after the use of IL-1β/TNFα, indicating that the synthesis of new extracellular matrix is not stimulated by small molecule inhibition. In addition, small molecules seem to have a repressive effect on bone growth, indicating a combined inhibitory effect on differentiation. These findings implicate the involvement of WNT/β-catenin signaling in the IL-1β/TNFα-induced effect on catabolic genes, but not in the effect on cartilage anabolic genes. In skeletal development, low levels of β-catenin are thought to promote chondroprogenitor differentiation, whereas, in later stages, high levels of β-catenin promote chondrocyte hypertrophic differentiation and subsequent endochondral ossification [35-37]. Based on these findings, inhibition of WNT/β-catenin signaling could be expected to induce cartilage matrix formation. However, low levels of WNT/β-catenin signaling seem not to have a stimulating effect on extracellular matrix formation after IL-1β- and TNFα-induced cartilage degradation. Other pathways, such as the MAPK/ERK pathway (mitogen-activated protein kinase and extracellular signal-regulated kinase) [38] and the nuclear factor κB pathway [39], were suggested to regulate the IL-1β-induced inhibition of gene expression of *ACAN *and *COL2A1*. Furthermore, immunofluorescence staining of β-catenin indicated that PKF115-584 might stabilize β-catenin in the cytosol, allowing for β-catenin to exert alternative effects such as direct binding to SOX9 and sequestering of SOX9 in the cytoplasm, thereby inhibiting expression of matrix genes.

Both PKF115-584 and PKF118-310 inhibit WNT/β-catenin signaling by blocking the binding of β-catenin to the transcription factor TCF4 [23]. We found differential effects of PKF115-584 and PKF118-310 on IL-1β/TNFα-induced cartilage degradation, which might be due to the fact that PKF115-584 inhibits translocation of β-catenin to the nucleus upon LiCl stimulation without affecting the basal amount of β-catenin, whereas PKF118-310 reduced both. In addition, CGP049090, which blocks the binding of β-catenin to TCF4 and Lef1 [24], was not as effective as PKF115-584. This might be due to the fact that PKF115-584 not only blocks binding of β-catenin to TCF4 but also disrupts binding of TCF4 to DNA [24].

## Conclusion

This study provides evidence for the involvement of WNT/β-catenin signaling in MMP-mediated cartilage degradation induced by IL-1β and TNFα. Furthermore, we show that WNT/β-catenin signaling is not involved in the repressive effects of IL-1β and TNFα on cartilage matrix proteins such as ACAN and COL2A1. Instead, we provide evidence that WNT/β-catenin signaling may be directly involved in the regulation of the expression of these extracellular matrix proteins via an as yet unknown mechanism.

## Abbreviations

ACAN: Aggrecan; cDNA: Complementary deoxyribonucleic acid; Col2a1: Collagen type 2a1; FZD: Frizzled; GAPDH: Glyceraldehyde 3-phosphate dehydrogenase; GSK3β: Glycogen synthase kinase 3β; IL-1β: Interleukin 1β; Lef1: Lymphoid enhancer-binding factor 1; LiCl: Lithium chloride; LRP: low-density lipoprotein receptor-related protein; MMP: Matrix metalloproteinase; mRNA: Messenger ribonucleic acid; OA: Osteoarthritis; RA: Rheumatoid arthritis; TCF4: Transcription factor 4; TNFα: Tumor necrosis factor α

## Competing interests

The authors declare that they have no competing interests.

## Authors' contributions

EL performed luciferase and MTT assays, analyzed data and drafted the manuscript. RM performed immunofluorescent staining for β-catenin. Both EL and RM performed *ex vivo *experiments. MK and CB contributed extensively to the discussion of experimental design and data interpretation. All authors read and approved the final manuscript.

## Supplementary Material

Additional file 1 **Figure S1 Small molecule inhibitors do not affect mouse fetal metatarsals**. (A) No morphological changes were found in metatarsals treated with small molecules PKF115-584, PKF118-310 or CGP049090 at a concentration of 1.0 µM. A representative picture of two independent experiments is shown. Scale bar represents 500 µm. (B) No significant differences were found in the bone length when metatarsals were treated with small molecules. Data are means with 95% confidence intervals of three independent experiments. Click here for file
