# Supplementary figures and images for: Small molecule inhibitors of WNT/β-catenin signaling block IL-1β- and TNFα-induced cartilage degradation
Source: Arthritis Res Ther. 2013 Aug 21;15(4):R93. doi: 10.1186/ar4273 (PMC3978727; doi:10.1186/ar4273)

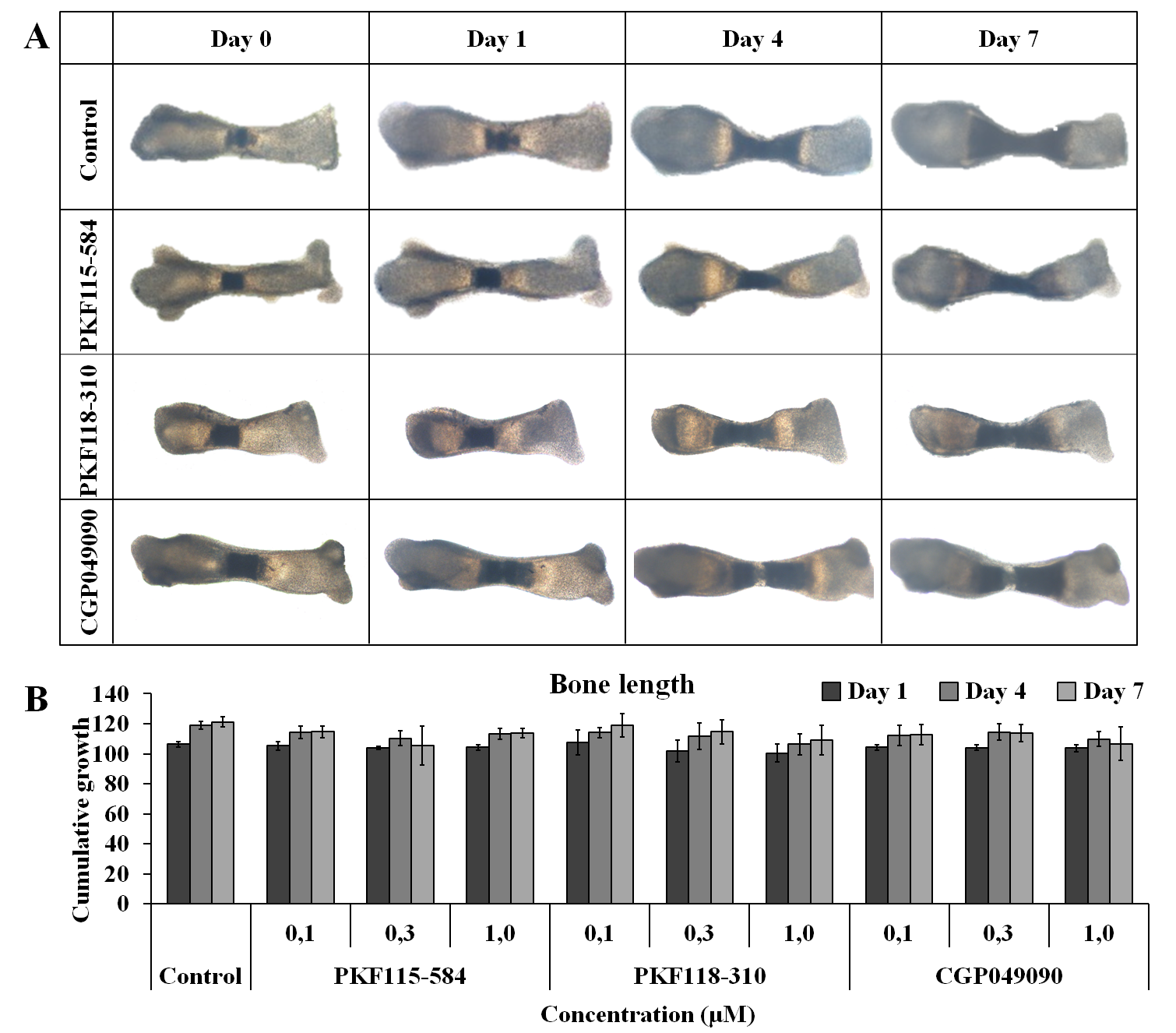

Supplement: Additional file 1 — Figure S1 Small molecule inhibitors do not affect mouse fetal metatarsals. (A) No morphological changes were found in metatarsals treated with small molecules PKF115-584, PKF118-310 or CGP049090 at a concentration of 1.0 µM. A representative picture of two independent experiments is shown. Scale bar represents 500 µm. (B) No significant differences were found in the bone length when metatarsals were treated with small molecules. Data are means with 95% confidence intervals of three independent experiments. [file ar4273-S1.PNG]
